# Influencing factors on cardiac structure and function beyond glycemic control in patients with type 2 diabetes mellitus

**DOI:** 10.1186/1475-2840-12-38

**Published:** 2013-02-27

**Authors:** Ryoko Ichikawa, Masao Daimon, Tetsuro Miyazaki, Takayuki Kawata, Sakiko Miyazaki, Masaki Maruyama, Shuo-Ju Chiang, Hiromasa Suzuki, Chiharu Ito, Fumihiko Sato, Hirotaka Watada, Hiroyuki Daida

**Affiliations:** 1 Department of Cardiovascular Medicine, Juntendo University School of Medicine, Tokyo, Japan; 2 Department of Metabolism & Endocrinology, Juntendo University School of Medicine, Hongo 2-1-1, Bunkyo-ku, Tokyo 113-8421, Japan

**Keywords:** Diabetes mellitus, Echocardiography, Cardiac function

## Abstract

**Background:**

We hypothesized that clinical factors other than glycemic control may influence abnormal cardiac function in patients with type 2 diabetes mellitus (T2DM). We aimed to investigate the independent factors for abnormal cardiac function among clinical factors in T2DM.

**Methods:**

We studied 148 asymptomatic patients with T2DM without overt heart disease. Echocardiographic findings were compared between diabetic patients and 68 age-matched healthy subjects. Early (E) and late (A) diastolic mitral flow velocity and early diastolic mitral annular velocity (e’) were measured for assessing left ventricular (LV) diastolic function. We evaluated insulin resistance, non-esterified fatty acid, high-sensitive CRP, estimated glomerular filtration rate, waist/hip ratio, abdominal visceral adipose tissue (VAT), subcutaneous adipose tissue (SAT) and other clinical characteristics in addition to glycemic control. VAT and SAT were quantified by computed tomography.

**Results:**

In T2DM, E/A and e’ were significantly lower, and E/e’, left atrial volume and LV mass were significantly greater than in control subjects. In multivariate liner regression analysis, VAT was an independent determinant of left atrial volume (β =0.203, p=0.011), E/A (β =−0.208, p=0.002), e’ (β =−0.354, p<0.001) and E/e’ (β=0.220, p=0.003). Age was also an independent determinant, whereas fasting plasma glucose and hemoglobin A1c levels were not. In addition to systolic blood pressure, waist-hip ratio (β=0.173, p=0.024) and VAT/SAT ratio (β=0.162, p=0.049) were independent determinants of LV mass.

**Conclusion:**

Excessive visceral fat accompanied by adipocyte dysfunction may play a greater role than glycemic control in the development of diastolic dysfunction and LV hypertrophy in T2DM.

## 

Diabetes mellitus may cause myocardial injury even in the absence of coronary artery disease, hypertension or valvular disease [1]. This cardiac dysfunction increases the risk of heart failure and subsequent mortality independently of underlying coronary artery disease and other cardiovascular risk factors [2-4]. Although the mechanisms of myocardial injury in diabetes mellitus are complex, several studies have identified diastolic dysfunction and left ventricular (LV) hypertrophy as major characteristics of abnormal cardiac function and structure in diabetes mellitus using echocardiography, even in the absence of hypertension [5-8], Using animal models, many previous investigations have documented possible mechanisms underlying myocardial injury in diabetic mellitus [1,9]. However, the pathophysiology of diabetic myocardial injury has been still unclear in the clinical setting. Not only glycemic control, but many other factors including hyperinsulinemia [10], increased fatty acids [11], inflammation [12], renal function [13] and myocardial steatosis [14] have been postulated to contribute to the development of abnormal function and structure in diabetic mellitus. Nevertheless, the independent influence of these factors on cardiac functional pmeters beyond glycemic control has not been adequately evaluated in humans. In addition, few studies [15-17] have included a control group, and comparison with age-matched controls is essential to evaluate LV diastolic dysfunction and hypertrophy because even healthy subjects > 60 years old may have significant diastolic dysfunction [18,19].

Recently, visceral fat accumulation has gained attention as playing an important role in the development and pathophysiology of type 2 diabetes mellitus (T2DM) [20,21]. Excessive visceral fat is closely associated with adipocyte dysfunction accompanied by increased inflammatory cytokine secretion and reduced anti-inflammatory adiponectin secretion, which can lead to cardiac and endothelial dysfunction [22-24]. Thus, we hypothesized that visceral fat accumulation may be associated with abnormal cardiac function and structure in T2DM.

The aims of our study were the following: (1) to clarify if diastolic dysfunction and LV hypertrophy are characteristics of abnormal cardiac function and structure in T2DM in comparison with age-matched healthy controls; and (2) to investigate the independent factors for diastolic dysfunction and LV hypertrophy among clinical factors including glycemic control, blood pressure, insulin resistance, fatty acid and visceral fat.

## Methods

### Study population

A total of 148 consecutive asymptomatic patients with T2DM and without cardiovascular disease, who were admitted to our institution (Juntendo University Hospital Tokyo, Japan) for diabetic educational program between January 2010 and May 2012, were prospectively enrolled in this study. Patients were included if they met the following inclusion criteria: no symptoms and history of heart disease, LV ejection fraction (LVEF) > 50%, absence of regional LV wall motion abnormalities, and clinically stable. Exclusion criteria were coronary artery disease, congenital heart disease, atrial fibrillation, significant heart valve disease, renal failure (serum creatinine > 2.0 mg/dl) and type 1 diabetes mellitus. In addition, 68 age-matched healthy controls without hypertension, dyslipidemia or diabetes mellitus served as a control group. The study protocol was approved by the Institutional Review Board of Juntendo University Hospital.

### Clinical data and echocardiographic measurements

This study was a prospective cohort study. The diagnoses of hypertension, dyslipidemia, coronary artery disease and cerebrovascular accident were assessed by the treating physician. Standard comprehensive two-dimensional and Doppler echocardiographic examinations were performed using commercially available systems. As indicators of obesity, body mass index was calculated from height and weight and the waist-hip ratio was calculated from the waist and hip circumference.

### Conventional, 2D and Doppler echocardiography

In all patients and control subjects, cardiac chamber quantification by 2D echocardiography was performed according to guidelines provided by the American Society of Echocardiography [25]. LV diameters were measured using 2D echocardiography according to the recommended criteria. The thickness of the intraventricular septum and the LV posterior wall were measured at end-diastole. LV mass was calculated using diastolic measurements of LV diameter and wall thickness on 2D echocardiography according to the formula recommended by the American Society of Echocardiography [25]. LV end-diastolic volume and end-systolic volume were determined from the apical views using modified biplane Simpson’s method. LV ejection fraction was calculated by the following equation: 100 × (end-diastolic volume - end-systolic volume)/end-diastolic volume. Each pmeter was indexed for body surface area (BSA), when appropriate.

For assessing conventional diastolic pmeters, left atrial volume was measured by Simpson’s biplane method (indexed to body surface area), and mitral inflow velocities were determined by pulsed Doppler and tissue Doppler imaging. The peak early (E) and late (A) diastolic velocity, the deceleration time from the peak of the early diastolic wave to baseline (DCT) and the E/A ratio were assessed from the mitral inflow velocity pattern. The mitral annular motion velocity was recorded at the medial mitral annulus site in the apical 4-chamber view by pulsed tissue Doppler echocardiography. Peak early (e’) and late (a’) diastolic velocity of the annulus were measured and the ratio of peak early diastolic transmitral flow velocity to annular velocity (E/e’) was calculated [26]. Furthermore, according to previous reports [26,27], we classified diastolic function in all subjects into one of four categories (normal, grade I, grade II or grade III) to compare the prevalence of diastolic dysfunction between healthy subjects and diabetic patients.

### Laboratory measurements

Blood samples for determination of serum creatinine, fasting blood sugar, glycosylated hemoglobin, immunoreactive insulin, C-peptide immunoreactivity, N-terminal pro B-type natriuretic peptide (NT-pro BNP) and lipid profiles (triglycerides, high-density lipoprotein cholesterol and low-density lipoprotein cholesterol) were drawn in patients with T2DM. Moreover, the homeostasis model assessment ratio (HOMA-R) index was calculated as fasting plasma glucose × fasting plasma insulin/22.5 for assessing insulin resistance [28]. Non-esterified fatty acid (NEFA) was measured since it has been suggested to play a critical role in triggering the development of cellular insulin resistance and myocardial contractile dysfunction [11]. In addition, high-sensitive C-reactive protein (hsCRP) was measured as a marker of inflammation. Estimated glomerular filtration rate (eGFR) was determined based on the new Japanese coefficient-modified Modification of Diet in Renal Disease (MDRD) study equation [29]. The formula is as follows: eGFR=194 × serum creatinine (SCr)^-1.094^ × age-0.287, where age is in years, SCr is in mg/dL and GFR is in mL/min/1.73 m^2^ body surface area. The product of this equation was multiplied by a correction factor of 0.739 in women.

### Abdominal fat area

VAT and subcutaneous adipose tissue (SAT) were measured quantitatively by computed tomography (CT) (Aquilion64, Toshiba, Tokyo, Japan), as previously reported [30]. Briefly, an axial CT scan at the level of the umbilicus was obtained for each participant using an electron beam CT scanner (commercial available software). Planimetric measurements at the level of the umbilicus have been shown to correlate well with volumetric quantification of VAT (r=0.81 in men and r=0.85 in women, p<0.001) and SAT (r=0.95 in men and r=0.85 in women, p<0.001) [30]. The images generated were transferred to a workstation and analyzed using commercial software. The coefficients of variation between two observers analyzing the same VAT and SAT images (n=30) ranged from 0.6% to 14.2% and from 0.1% to 7.3%, respectively. Both VAT and SAT was indexed for body surface area as well as echocardiographic pmeters.

### Statistical analysis

All statistical analyses were performed using SPSS version 17.0 (SPSS, Inc., Chicago, IL). Data are expressed as mean ± standard deviation (SD). The relationships between variables were assessed using Pearson’s correlation coefficient. We compared baseline characteristics and echo pmeters between the control group and patients with T2DM using an unpaired t-test. Categorical data were compared between the two groups using a chi-square test. When we found echocardiographic pmeters that were different between the diabetics and controls, Pearson’s linear correlation analysis was used to determine the correlations between these echocardiographic pmeters and the clinical data in just the patients with T2DM. Multiple linear regression analysis was also performed using the clinical data to evaluate the independent determinants of these echocardiographic pmeters in the T2DM patients. A log-transformation was used to normalize the distributions of triglyceride, HOMA-R and NT-proBNP level. A P-value <.05 was considered statistically significant.

## Results

Table 1 shows the baseline characteristics of the study population. The study population consisted of 68 healthy controls and 148 patients with T2DM (total, 216). In patients with T2DM, body surface area, body mass index, blood pressure and heart rate were significantly higher than in normal controls. In the group with T2DM, 48% had hypertension and 63% had dyslipidemia.

**Table 1:** Baseline characteristics of the study population

**Variable**	**Controls (n = 68)**	**Patients with T2DM (n = 148)**	**p value**
Demographics			
Age, year	56 ± 11	58 ± 12	0.16
Men/Women	45/23	95/53	0.72
Systolic BP, mm Hg	118 ± 11	126 ± 17	<0.001
Diastolic BP, mm Hg	71 ± 7	68 ± 10	<0.001
Heart rate, bpm	62 ± 9	71 ± 10	<0.001
Body mass index, kg/m^2^	22.6 ± 2.6	26.1 ± 5.5	<0.001
Body surface area, m^2^	1.66 ± 0.16	1.76 ± 0.38	0.002
Disease duration, months	0	121 ± 93	
Waist/Hip ratio	-	0.89 ± 0.20	
Comorbidity			
Hypertension, n (%)	0	71 (48%)	
Dyslipidemia, n (%)	0	93 (63%)	
Cerebral vascular disease, n (%)	0	9 (6%)	
ASO, n (%)	0	4 (3%)	
Smoking, n (%)	-	68 (46%)	
Medications	0	101 (68%)	
Sulfonylureas, n (%)	0	57 (38%)	
Biguanide, n (%)	0	56 (38%)	
α-glucocitase inhibitor, n (%)	0	37 (25%)	
Pioglitazone, n (%)	0	22 (15%)	
Insulin, n (%)	0	45 (30%)	
DPP-4 inhibitor, n (%)	0	21 (14%)	
GLP-1 analog, n (%)	0	4 (3%)	

*BP*, blood pressure; *ASO*, atherosclerotic obliterance; *DPP-4*, Dipeptidyl peptidase-4.

Data are expressed as mean ± SD it as number (percentage).

### Comparison of echocardiographic pmeters between the two groups

**Figure 1:**
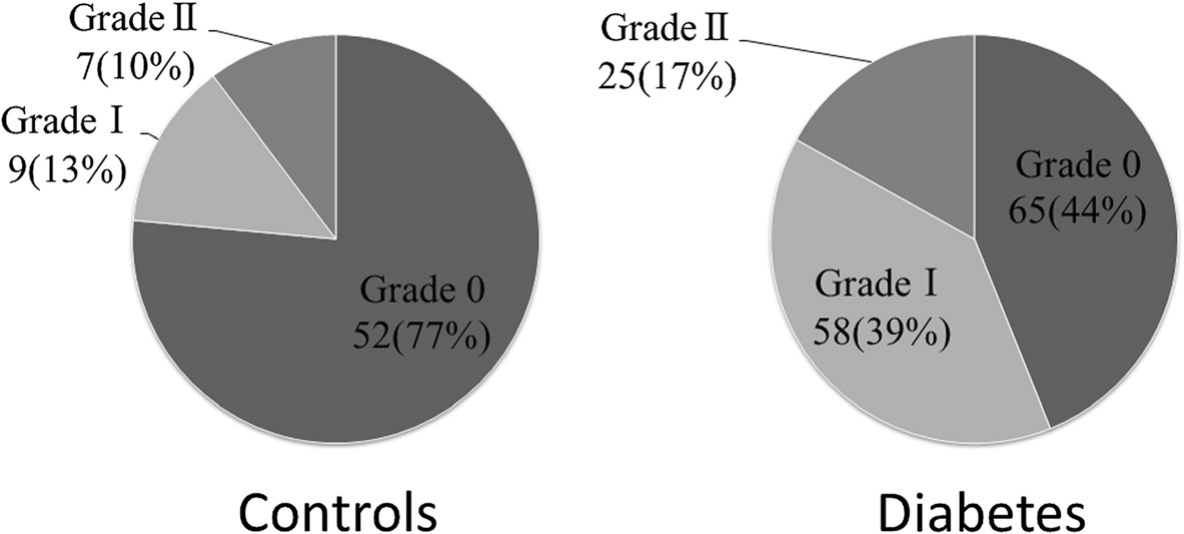
Distribution of diastolic dysfunction in age-matched healthy subjects and patients with T2DM.

Table 2 shows differences in echocardiography pmeters between the two groups. Patients with T2DM had a greater LV mass index than normal controls. LV mass index of control subjects, diabetic patients without hypertension, and diabetic patients with hypertension, were 70±12, 79±18, and 83±22 g/m^2^, respectively. LV mass index of both diabetic patients with and without hypertension were increased as compared with control subjects (p<0.01 and p<0.001, respectively) as shown in previous studies [5-8]. Patients with T2DM also had a significantly larger left atrial volume index, lower e’ and E/A, and higher E/e’, indicating LV diastolic dysfunction, as previously reported [5-8]. Whereas 23% of the age-matched healthy controls had diastolic dysfunction with advancing age, 56% of patients with T2DM had diastolic dysfunction, indicating a higher prevalence of diastolic dysfunction in the diabetic population (Figure 1). None of the subjects were classified into grade III diastolic dysfunction.

**Table 2:** Comparison of echocardiographic pmeters between normal controls and patients with T2DM

	**Controls (n = 68)**	**Patients with T2DM (n = 148)**	***p *value**
LV Mass index, g/m^2^	70 ± 12	81 ± 20	<0.001
LVEDV index, ml/m^2^	49 ± 12	51 ± 12	0.155
LVESV index, ml/m^2^	17 ± 4.8	17 ± 5.0	0.450
LV ejection fraction, %	64 ± 5	68 ± 5	<0.001
LA volume index, ml/m^2^	22 ± 8	24 ± 8	0.041
E/A ratio	1.1 ± 0.4	0.9 ± 0.3	<0.001
DCT time, msec	205 ± 46	206 ± 48	0.924
e’, cm/sec	8.2 ± 2.5	6.5 ± 1.8	<0.001
a’, cm/sec	9.4 ± 1.9	6.4 ± 1.8	0.783
E/e’ ratio	8.3 ± 2.1	9.7 ± 3.3	0.001

*LVEDV*, left ventricular end-diastolic volume; *LVESV*, left ventricular end-systolic volume; *LA*, left atrial.

### Determinants of the abnormal echocardiographic pmeters in T2DM

Laboratory data and visceral fat area measured by CT scanning are shown in Table 3. Among the clinical factors in Tables 1 and 3, we determined which factors were associated with the abnormal echocardiographic pmeters shown in Table 2. The LV mass index had a significant correlation with systolic blood pressure, waist/hip ratio, low-density cholesterol level and VAT/SAT ratio (Table 4). Multivariate liner regression analysis found that systolic blood pressure, waist/hip ratio and VAT/SAT ratio were independent factors associated with LV mass index (Table 5).

**Table 3:** Laboratory data and adipose tissue area with computed tomography in patients with T2DM

	**Patients with T2DM (N=148)**
Laboratory data	
Creatinine, mg/dl	0.92 ± 0.17
eGFR, ml/min	83 ± 26
Triglyceride, mg/dl	139 ± 100
HDL cholesterol, mg/dl	50 ± 15
Total cholesterol, mg/dl	198 ± 34
F-LDL cholesterol, mg/dl	119 ± 32
Fasting plasma glucose, mg/dl	138 ± 38
Hemoglobin A1c, %	7.9 ± 1.4
NEFA, μEq/L	613 ± 202
IRI, μg/dl	8.7 ± 7.0
HOMA-R	2.9 ± 2.7
hsCRP, mg/dl	0.190 ± 0.357
NT-proBNP, pg/ml	36.2 ± 55.3
CPR, mg/dl	2.8 ± 9.6
Fat areas determined by CT scan	
Total adipose tissue/BSA, cm^2^/m^2^	169.4 ± 74.9
Visceral adipose tissue/BSA, cm^2^/m^2^	68.3 ± 30.6
Subcutaneous adipose tissue/BSA, cm^2^/m^2^	101.1 ± 54.3

*eGFR*, estimated glomerular filtration rate; *HDL*, high-density lipoprotein; *F-LDL*, low-density lipoprotein calculated by Friedewald; *NEFA*, non-esterified fatty acid; *IRI*, immunoreactive insulin; *HOMA*, homeostasis model assessment; *hsCRP*, high-sensitivity C reactive protein; *NT-pro BNP*, N-terminal pro b-type natriuretic peptide; *CPR*, C-peptide immunoreactivity; *CT*, computed tomography; *BSA*, body surface area.

**Table 4:** Pearson’s correlation between echocardiographic pmeters and clinical data

	**LV mass index**		**LA volume index**		**E/A**		**e’**		**E/e’**	
	***r***	***p***	***r***	***p***	***r***	***p***	***r***	***p***	***r***	***p***
Female gender	0.050	n.s.	-0.88	0.290	-0.168	0.040	-0.255	0.002	0.265	0.001
Age, year	0.130	n.s.	0.235	0.004	-0.495	<0.001	-0.464	<0.001	0.300	<0.001
Disease duration	0.046	n.s.	0.206	0.012	-0.167	0.041	-0.190	0.021	0.076	n.s.
Systolic BP, mmHg	0.202	0.013	0.123	n.s.	-0.202	0.014	-0.156	n.s.	0.103	n.s.
Diastolic BP, mmHg	0.075	n.s.	-0.119	n.s.	0.055	n.s.	-0.036	n.s.	-0.059	n.s.
Body mass index, kg/m^2^	0.092	n.s.	0.060	n.s.	0.000	n.s.	-0.064	n.s.	0.042	n.s.
Waist/Hip ratio	0.173	0.035	-0.003	n.s.	-0.115	n.s.	-0.114	n.s.	0.094	n.s.
Triglyceride, mg/dl	0.084	n.s.	-0.048	n.s.	-0.056	n.s.	-0.016	n.s.	0.090	n.s.
HDL cholesterol, mg/dl	-0.006	n.s.	-0.143	n.s.	0.001	n.s.	0.071	n.s.	0.036	n.s.
F-LDL cholesterol, mg/dl	-0.187	0.022	0.012	n.s.	0.071	n.s.	0.064	n.s.	-0.049	n.s.
Fasting plasma glucose, mg/dl	0.007	n.s.	-0.004	n.s.	0.074	n.s.	-0.021	n.s.	0.127	n.s.
HbA1c, %	0.037	n.s.	0.072	n.s.	0.061	n.s.	0.097	n.s.	0.063	n.s.
IRI, ug/dl	-0.111	n.s.	-0.070	n.s.	-0.011	n.s.	-0.093	n.s.	0.043	n.s.
HOMA-R	-0.037	n.s.	-0.057	n.s.	0.008	n.s.	-0.121	n.s.	0.103	n.s.
eGFR, ml/min	0.015	n.s.	-0.040	n.s.	0.310	<0.001	0.266	0.001	-0.092	n.s.
NEFA, μEq/L	-0.022	n.s.	0.086	n.s.	0.051	n.s.	0.075	n.s.	-0.092	n.s.
hsCRP, mg/dl	-0.107	n.s.	-0.143	n.s.	0.135	n.s.	-0.028	n.s.	0.050	n.s.
Hypertensions	0.105	n.s.	0.198	0.016	-0.128	n.s.	-0.340	<0.001	0.218	0.008
VAT/BSA, cm^2^/m^2^	0.155	n.s.	0.197	0.016	-0.205	0.012	-0.320	<0.001	0.217	0.008
SAT/BSA, cm^2^/m^2^	0.003	n.s.	0.114	n.s.	-0.059	n.s.	-0.177	0.031	0.186	0.023
VAT/SAT ratio	0.164	0.045	0.022	n.s.	-0.098	n.s.	-0.087	n.s.	-0.034	n.s.

For abbreviations, see footnotes to Tables 1, 2 and 3.

**Table 5:** Multivariate linear regression analysis between the echocardiographic pmeters and clinical data

	**LV mass index**		**LA volume index**		**E/A**		**e’**		**E/e’**	
	**β**	***p***	**β**	***p***	**β**	***p***	**β**	***p***	**β**	***p***
Female gender							−0.211	0.009	0.213	0.006
Age, year			0.245	0.003	−0.524	<0.001	−0.464	<0.001	0.245	<0.002
sBP, mmHg	0.205	0.013								
Waist/Hip ratio	0.173	0.024								
F-LDL cholesterol, mg/dl										
eGFR, ml/min										
Hypertension							−0.324	<0.001	0.199	0.006
VAT/BSA, cm^2^/m^2^			0.203	0.011	−0.208	0.002	−0.354	<0.001	0.220	0.003
VAT/SAT ratio	0.162	0.049								

For abbreviations, see footnotes on Tables 1, 2 and 3.

**Figure 2:**
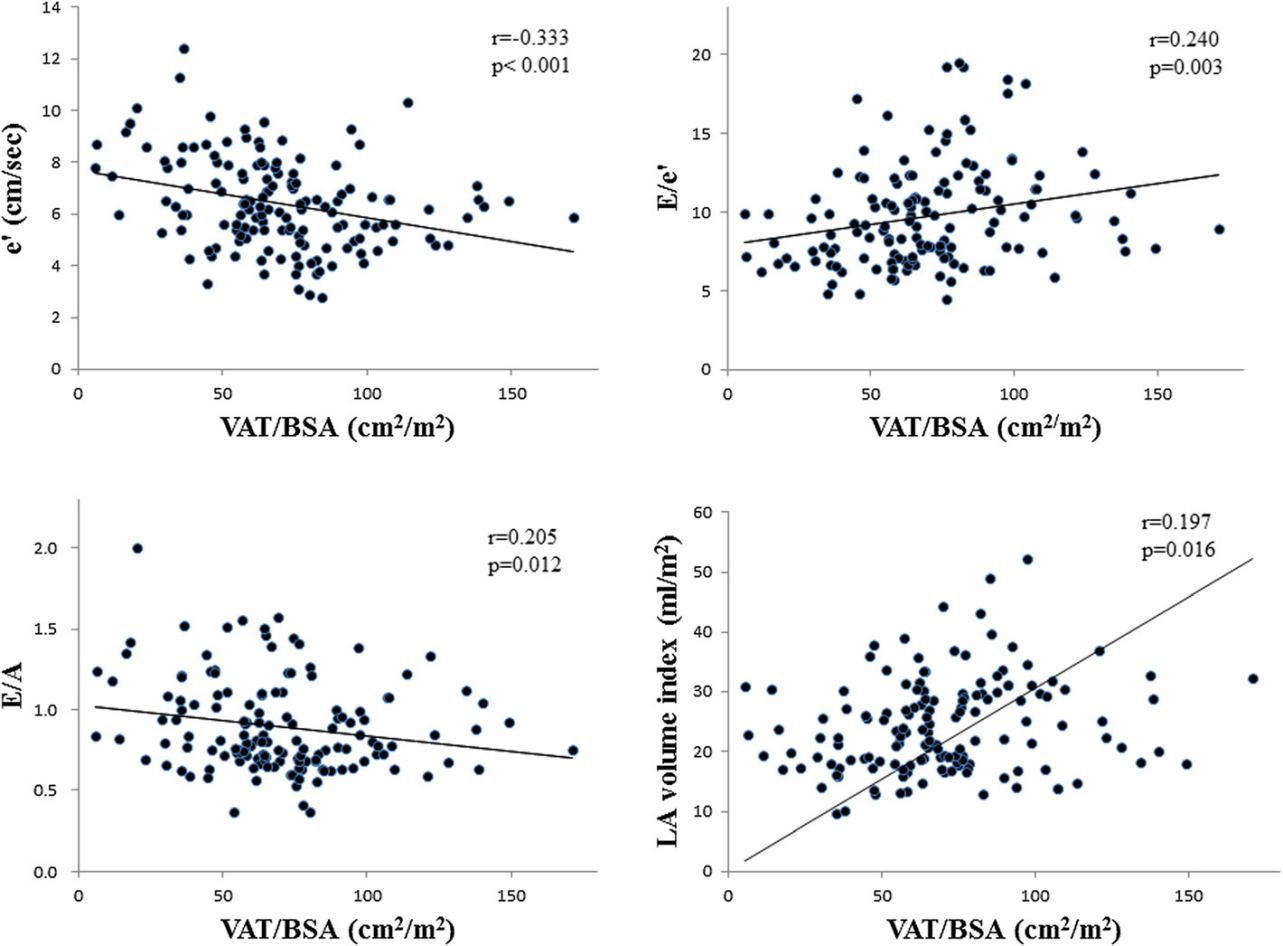
**Correlations between VAT and echo pmeters (E/A, e’, E/e’, LA volume index).** VAT was significantly correlated with these diastolic pmeters. Pearson’s correlation coefficients and significance are shown.

The correlation between clinical factors and the diastolic pmeters are also shown in Table 4. These diastolic pmeters were mainly correlated with gender, age, hypertension and VAT, whereas E/A and e’ were also correlated with decreased eGFR. In multivariate linear regression analysis, age was the strongest independent determinant of left atrial volume, E/A, e’ and E/e’ among all the clinical variables. Furthermore, VAT was also an independent determinant of these four diastolic pmeters (Table 5, Figure 2), indicating excess VAT may be independently associated with diastolic dysfunction in T2DM. As for e’ and E/e’, female gender and hypertension were independent determinants. Furthermore, there were positive correlations between VAT and log triglyceride (r=0.376, *p*<0.001), IRI (r=0.393, *p*<0.001) and log HOMA-R (r=0.363, *p*<0.001).

## Discussion

In the current study, we found that LV diastolic dysfunction and hypertrophy were more prominent in patients with T2DM than in age-matched healthy controls, as previously reported [5-8]. Furthermore, we identified the independent factors associated with LV diastolic dysfunction and hypertrophy. Gender, age, hypertension and VAT were all independently associated with diastolic dysfunction, and the waist/hip and VAT/SAT ratios were independently associated with increased LV mass, in addition to systolic blood pressure. Of note, our study is the first to show that excess VAT is more important than glycemic control, insulin resistance, renal function and other clinical factors in abnormal cardiac structure and function in T2DM.

Many previous studies have demonstrated a high prevalence of LV diastolic dysfunction and hypertrophy in patients with T2DM [5-8]. However, few studies have included a control group [15-17]. Diastolic dysfunction progresses with age even in a healthy population, and diastolic dysfunction is a common phenomenon in healthy individuals over 60 years old [18,19]. Thus, it is important to compare diabetics with age-matched control subjects when evaluating the prevalence of diastolic dysfunction. In this study, a higher prevalence of diastolic dysfunction, in addition to increased LV mass, was found in T2DM compared with control subjects. The 23% prevalence of diastolic dysfunction in the control subjects was probably due to aging.

Myocardial injury in T2DM has a complicated pathophysiology [1,9], and many factors, such as hyperinsulinemia [10], increased fatty acid [11], inflammation [12], renal function [13] and myocardial steatosis [14], have been postulated to be involved in its development. However, most previous clinical studies of diabetic cardiac dysfunction examined only glycemic control and other common cardiovascular risk factors, such as hypertension or obesity [5-8,15-17]. Furthermore, some recent review articles that performed meta-analysis of multiple trials reported that intensive glycemic control does not inevitably result in a reduction of cardiovascular events and mortality [31,32]. Thus, we hypothesized that some factors other than glycemic control may play an important role in the development of abnormal cardiac structure and function in T2DM. In this study, we investigated the association between echocardiographic pmeters in T2DM and several common risk factors, as well as factors that have not been examined before in the clinical setting. Importantly, excess VAT in our study, but not glycemic control, was found to be independently associated with diabetic diastolic dysfunction, in addition to gender, age and hypertension. Visceral fat accumulation might be a potential therapeutic target to improve cardiac dysfunction in T2DM rather than glycemic control.

Visceral adipose tissue is an important functional organ which abundantly secretes adipocytokines [33]. In the lean state, small adipocytes efficiently store fatty acids as triglyceride and have the ability to inhibit the inflammatory state. In particular, high levels of adiponectin in the lean state have anti-inflammatory effects [34]. On the other hand, increased visceral adipose tissue secrete various inflammatory cytokines, including tumor necrosis factor-α (TNF-α), interleukin-6 (IL-6), IL-8 and MCP-1. These adipocytokines are assumed to contribute to changes in cardiac structure and function [22-24].

Increased inflammatory cytokines such as IL-6, IL-8 and MCP-1 have been shown to be significant indicators of a greater degree of heart failure with preserved EF [35]. High plasma levels of TNF-α and IL-6 might cause cardiac diastolic dysfunction by decreasing diastolic calcium reuptake in myocytes [36]. Studies in vitro showed that cardiac-restricted overexpression of TNF-α induces myocardial fibrosis and diastolic dysfunction [37]. Furthermore, low plasma adiponectin had an association with early diastolic dysfunction in patients with heart failure [24]. Taken together, these studies suggest that visceral fat accumulation may influence diastolic function through changes in inflammatory and anti-inflammatory adipocytokines. Furthermore, dysfunctional adipose tissue produces angiotensinogen and angiotensin II, which induces systemic hypertension [34]. In this study, 49% of patients in the diabetes mellitus group had hypertension. Although activation of the renin-angiotensin system in diabetes mellitus per se is associated with increased oxidative damage and cardiomyocyte apoptosis and necrosis in the diabetic heart [38], hypertension due to visceral adiposity may also influence diastolic function in T2DM. On the other hand, hsCRP, an inflammatory maker, was not directly related to cardiac dysfunction in the present study, whereas we found a significant correlation between hsCRP and VAT (*r*=0.281, *p*<0.001). HsCRP can reflect inflammatory processes in various organs and is not only the mechanism by which VAT causes diastolic dysfunction. Presumably other inflammatory cytokines and anti-inflammatory cytokines relate to visceral fat accumulation and are also significant determinant factors for cardiac dysfunction in T2DM.

Age-related decline in LV diastolic function even in a healthy population has been widely reported using conventional and tissue Doppler methods [18,19]. Gender differences in diastolic function have also been reported previously [19]. Epidemiological and clinical studies have consistently demonstrated that elderly women in the general population were most likely to have diastolic heart failure [39]. Menopausal hormone status has been suggested to be one of the factors to account for gender differences in diastolic dysfunction [40], although the precise mechanism has not been clarified.

As for LV hypertrophy, the waist/hip and VAT/SAT ratios were found to be independent determinants in addition to systolic blood pressure. The waist/hip ratio, an indicator of obesity, was reported to be a predictor of cardiovascular mortality [41]. Abnormal fat distribution expressed as the VAT/SAT ratio was associated with the development of insulin resistance and atherosclerosis [42]. In this study, we could not determine the reason why the waist/hip and VAT/SAT ratios, but not the absolute amount of VAT, were independently related to increased LV mass. However, there is no doubt that visceral fat and obesity play some roles in the development of LV hypertrophy based on mechanisms similar to those of diastolic dysfunction in T2DM.

### Study limitations

While we included asymptomatic patients without overt cardiovascular disease, we did not completely exclude latent patients with coronary artery disease using stress testing or coronary angiography. We did not measure serum adipocytokine levels and could not evaluate adipose dysfunction. Further investigation is required to determine the direct association between VAT and the level of adipocytokine dysfunction in diabetic cardiomyopathy. We included patients with the comorbidity of hypertension in this study. Hypertension caused by activation of the renin-angiotensin system in diabetes mellitus plays an important role in the development of cardiac dysfunction in T2DM. In addition, patients with T2DM have high prevalence of hypertension in the real clinical setting and are more subject to LV hypertrophy caused by hypertension than those without diabetes [43]. These characteristics are important aspects of cardiac dysfunction in T2DM. Instead, we performed multiple regression analysis using clinical factors including history of hypertension and blood pressure to evaluate the independent determinants of LV mass and diastolic dysfunction, and VAT or VAT/SAT ratio was an independent determinant of cardiac structure and function in our population after adjustment for history of hypertension and blood pressure. However, hypertension per se causes diastolic dysfunction and LV hypertrophy, and further investigation in normotensive patients with T2DM is desirable to more accurately assess the mechanisms of cardiac abnormalities in T2DM. Moreover, further investigation in comparison with patients with hypertension but without T2DM would provide new insights to this issue. Finally, we measured only LV ejection fraction as an indicator of systolic function in this study. Ernande et al [17]. reported reduced systolic strain using speckle-tracking imaging in T2DM; therefore, further investigation of systolic dysfunction with speckle-tracking imaging would be of interest.

## Conclusion

This study confirms that diastolic dysfunction and LV hypertrophy are major characteristics of abnormal cardiac structure and function in T2DM. Among many clinical variables, excess VAT may play a significant role in the development of diastolic dysfunction and LV hypertrophy beyond glycemic control. Visceral fat accumulation would be a potential therapeutic target to improve cardiac dysfunction rather than glycemic control in T2DM.

## Competing interest

There is no relationship with any industry.

## Authors’ contributions

RI and MD have designed this study in whole and drafted this manuscript. MM, SM, SC, TK and HS have contributed to design the echo part in this study and interpret its results. CI and FS have contributed to design the visceral adipose tissue part in this study and interpret its results including laboratory data. HW has contributed to design this study in part and interpret the clinical data. TM has contributed to statistical analyses in this study. HD has revised this manuscript critically for important intellectual content and approved finally the manuscript submitted. All authors read and approved the final manuscript.

## Funding sources

This work was partially supported by a Grant-in-Aid for Scientific Research C (24500554) from the Japan Society for the Promotion of Science (Masao Daimon).
